# Allocating operating room resources to an acute care surgery service does not affect wait-times for elective cancer surgeries: a retrospective cohort study

**DOI:** 10.1186/1749-7922-9-21

**Published:** 2014-03-27

**Authors:** Ram Venkatesh Anantha, Dave Paskar, Kelly Vogt, Silvie Crawford, Neil Parry, Ken Leslie

**Affiliations:** 1 Division of General Surgery, Department of Surgery, Schulich School of Medicine and Dentistry, Western University, London, ON, Canada; 2 Division of Trauma and Acute Care Surgery, Department of Surgery, Los Angeles County and University of Southern California Medical Center, Los Angeles, CA, USA; 3 Executive Leadership (Patient Centred Care), London Health Sciences Centre, London, ON, Canada; 4 Trauma Program, London Health Sciences Centre, London, ON, Canada; 5 Division of Critical Care, Department of Medicine, Schulich School of Medicine and Dentistry, Western University, London, ON, Canada; 6 Victoria Hospital, London Health Sciences Centre, Room E2-217, 800 Commissioners Road East, London, ON N6A 5W9, Canada

**Keywords:** Acute care surgery, Cancer wait-times, Resource allocation, Health outcomes research

## Abstract

**Introduction:**

Acute care surgical services provide timely comprehensive emergency general surgical care while optimizing the use of limited resources. At our institution, 50% of the daily dedicated operating room (OR) time allocated to the Acute Care Emergency Surgery Service (ACCESS) came from previous elective general surgery OR time. We assessed the impact of this change in resource allocation on wait-times for elective general surgery cancer cases.

**Methods:**

We retrospectively reviewed adult patients who underwent elective cancer surgeries in the pre-ACCESS (September 2009 to June 2010) and post-ACCESS (September 2010 to June 2011) eras. Wait-times, calculated as the time between booking and actual dates of surgery, were compared within assigned priority classifications. Categorical and continuous variables were compared using chi-square and Mann–Whitney U tests respectively.

**Results:**

A total of 732 cases (367 pre-ACCESS and 365 post-ACCESS) were identified, with no difference in median wait-times (25 versus 23 days) between the eras. However, significantly fewer cases exceeded wait-time targets in the post-ACCESS era (p <0.0001). There was a significant change (p = 0.027) in the composition of cancer cases, with fewer breast cancer operations (22% versus 28%), and more colorectal (41% versus 32%) and hepatobiliary cancer cases (5% versus 2%) in the post-ACCESS era.

**Conclusion:**

These results suggest that shifting OR resources towards emergency surgery does not affect the timeliness of surgical cancer care. This study may encourage more centres to adopt acute care surgical services alongside their elective or subspecialty practices.

## Introduction

Acute care surgery (ACS) is a distinct surgical care model that provides dedicated comprehensive care for general surgical emergencies such as acute appendicitis, cholecystitis, bowel obstruction, perineal sepsis, and perforated viscus [1-3]. This model has proven to be an innovative and cost-effective strategy of delivering emergency surgical care to patients [1,3], resulting in significantly shorter wait-times for urgent and emergent operations [4-7], more efficient disposition from the emergency room [4-7], and considerably reduced hospital costs [5,8,9] in many centres. Surgeons also benefit from this model as it offers more predictable scheduling, reduced nocturnal workload, and enables them to focus on elective patient care or academic endeavours when they are not on call for ACS [1].

The local delivery and structure of ACS services can vary significantly from hospital to hospital, particularly in terms of the availability of dedicated ACS operating room (OR) time. Because of the financial constraints associated with a publicly-funded healthcare system, Canadian hospitals have typically funded dedicated ACS OR time by reallocating existing OR resources, rather than providing additional funding *de novo*. At the London Health Sciences Centre (LHSC) - Victoria Hospital, the Acute Care and Emergency Surgery Service (ACCESS) was established in July 2010 when the growing need for organized emergency general surgery coverage was recognized by the Division of General Surgery, the Emergency Department, and hospital leadership. In this model, a single staff surgeon suspends their elective practice while covering ACCESS for one week at a time (Monday to Monday), and their previously-allocated elective OR time for the week (15 hours) is subsumed into the daily dedicated ACCESS OR time. An additional 15 hours of OR time per week was funded by the South West Local Health Integration Network (the health authority responsible for administering public healthcare services within Southwestern Ontario) specifically for the ACCESS initiative, for a total of 30 hours per week dedicated to the service. The team is also assigned a full complement of housestaff.

In 2004, Ontario’s Ministry of Health and Long-Term Care (MOHLTC) implemented a Wait Time Strategy [10-13] to improve access to healthcare services for adult patients in five “key” populations, one of which was those requiring cancer surgery. Target wait-times were developed by Cancer Care Ontario (CCO) and the Surgical Access to Care and Wait Times Subcommittee [10,14], and provincial funding for centres providing surgical care for cancer patients was based on adherence to these suggested guidelines [10,13]. Since all the surgeons at LHSC who participate in ACCESS also perform cancer operations as part of their subspecialty practices, we sought to determine if the weekly suspension of one surgeon’s elective practice and diversion of their elective OR time for the week had a negative impact on wait-times for cancer surgeries.

## Methods

All clinical activity reviewed occurred at Victoria Hospital (VH), LHSC in London, Canada, which serves as a regional tertiary-care hospital and Level I trauma centre for Southwestern Ontario. The Division of General Surgery at VH is a diverse group of sub-specialists, including colorectal, hepatobiliary, endocrine, surgical oncology, trauma, and minimally invasive surgeons. All eight general surgeons at Victoria Hospital were involved with ACCESS during the study period, and performed oncological surgeries as part of their subspecialty practices, including thyroid, breast, colorectal, hepatobiliary (HPB), foregut (gastric and duodenal), endocrine, and melanoma surgery. Other surgical specialties, including plastic, orthopaedic, urologic, gynecologic, and head and neck surgery, also routinely perform cancer operations at VH.

Ethics approval for this single-centre retrospective cohort study was provided by the Western University Research and Ethics Board (REB Number 102988). The LHSC-VH operative database was queried for all elective cancer operations performed by all surgical specialties between September 1, 2009 and June 30, 2010 (pre-ACCESS) and between September 1, 2010 and June 30, 2011 (post-ACCESS). Cancer surgeries were defined as oncological operations booked electively. As part of the provincial Wait-Time Strategy initiative, all cancer operations were assigned a certain priority status by the surgeon at the time of booking based on the perceived urgency of the intervention (Table 1). Recommended wait-times for surgery are determined by the assigned priority and range from immediate (for patients with life-threatening malignancies; “P1” status) to 84 days (for patients with indolent tumours; “P4” status). Wait-times are defined as the length of time between the decision-to-treat date (defined as the date on which the surgeon and patient made the decision to pursue surgery) and the date that the operation was actually performed.

**Table 1:** Recommended target wait times (days) for cancer operations based on assigned priority category, as established by the Cancer Care Ontario sub-committee on cancer wait times

**Priority category**	**Clinical conditions**	Consult to decision-to-treat*	**Ready-to-treat to operation**
P1	Patients requiring surgery to remove known or suspected cancers that have immediately life-threatening conditions (e.g., airway obstruction, hemorrhage, neurological compromise)	Immediate	Immediate
P2	Patients diagnosed with very aggressive tumours, such as central nervous system (CNS) cancer	14	14
P3	All patients with known or suspected invasive cancer that does not meet the criteria of urgency category II or IV	14	28
P4	Patients diagnosed with indolent tumours	14	84

*From the date of the patient’s first visit to the operating surgeon for this specific problem until the decision-to-treat date. The decision-to-treat date is the date on which sufficient pre-treatment testing is complete, the physician can reasonably assume that the patient will be treated, and the patient has agreed to the treatment. By this date, sufficient assessment will have been completed in order to reasonably assume that the procedure will go ahead, and an operating room booking is requested.

All adults (age 18 and older) undergoing elective cancer surgery with curative intent and whose decision-to-treat and operation dates fell within the defined study periods were included. We excluded patients whose cases were booked in emergency or ACCESS OR time, and patients who were assigned a P1 priority status, since they required an imminent operation and thus were typically operated on non-electively. We also excluded patients who underwent surgery to remove benign or pre-malignant tumours, to correct or repair defects from previous cancer operations (reconstructive surgery), or to provide palliation.

Analyses were carried out on the basis of surgeries performed by general surgeons, as well as the overall patient population. Continuous variables were compared using the Mann–Whitney U-test. Categorical variables were compared using chi-square or Fisher’s exact tests where indicated. P-values less than 0.05 were considered statistically significant. Statistical analysis was performed using Graphpad Prism Version 5 (Graphpad, La Jolla, California).

## Results

We identified a total of 732 patients who underwent cancer surgery by the general surgeons at VH across the two study periods (Table 2). There were 365 elective cancer surgeries performed in the post-ACCESS, compared to 367 cases performed in the pre-ACCESS period. Overall, there was no difference in the median wait-times (25 versus 23 days) between the eras for elective general surgery cancer operations (p = 0.82).

**Table 2:** Distribution of elective cancer operations performed by general surgeons at Victoria Hospital, before (pre-ACCESS) and after (post-ACCESS) the implementation of ACCESS

**Variable**	**Pre-ACCESS**	**Post-ACCESS**	**Change, n (%)**	**P value**
Number of cases, n	367	365	-2 (-1)	-
Number of cases by priority, n (%)				<0.0001
P2	21 (6)	1 (0.3)	-20 (-95)	
P3	277 (75)	167 (46)	-110 (-40)	
P4	69 (19)	197 (54)	+128 (+185)	
Number of cases exceeding wait-time targets, n (%)				<0.0001
P2	13 (62)	0 (0)	-13 (-100)	
P3	92 (33)	41 (25)	-51 (-55)	
P4	2 (3)	2 (1)	0 (0)	
Median wait-times by priority, days (range)				0.94
P2	15 (2–29)	9 (N/A)	-6 (-40)	
P3	21 (0–90)	15 (0–90)	-6 (-29)	
P4	33 (6–92)	22 (0–90)	-11 (-33)	
Type of cancer, n (%)				0.027
Breast	104 (28)	79 (22)	-25 (-24)	
Colorectal	119 (32)	151 (41)	+32 (+27)	
Hepatopancreatobiliary	8 (2)	18 (5)	+10 (+125)	
Gastric	10 (3)	5 (1)	-5 (-50)	
Endocrine	100 (27)	94 (26)	-6 (-6)	
Lymph	1 (0)	0 (0)	-1 (-100)	
Soft-tissue sarcoma	6 (2)	8 (2)	+2 (+33)	
Skin carcinoma^1^	4 (1)	2 (1)	-2 (-50)	
Skin melanoma	15 (4)	7 (2)	-8 (-53)	

^1^Includes basal and squamous cell carcinoma.

The distribution of general surgery cancer cases by priority level was significantly different (p < 0.0001) between the eras: in the post-ACCESS period, P2 and P3 cases declined by 95% and 40%, respectively, while P4 cases rose by 185%. There was no significant change in wait-times for elective general surgery cancer cases pre- and post-ACCESS, according to priority status. However, the proportion of cases that exceeded assigned wait-time targets in the post-ACCESS era declined by 100% and 55% for P2 and P3 cases, respectively (p < 0.0001), while the proportion of P4 cases that exceeded wait-time targets did not change (Table 2). There was also a significant change in the type of cancer operated by general surgeons post-ACCESS: breast cancer, skin carcinoma, and skin melanoma cases declined by 24%, 50%, and 53%, respectively, whereas colorectal and hepatobiliary cases increased by 27% and 125%, respectively (p = 0.027).

There were 3309 cancer surgeries performed by non-general surgeon specialists at VH during the study periods (Table 3). There was a 4% reduction in the total number of cancer surgeries performed in the post-ACCESS era. The distribution of cancer cases by priority level was also significantly different post-ACCESS (p < 0.0001): P2 and P3 cases declined by 49% and 25%, respectively, while P4 cases rose by 62%. Furthermore, the number of cases that exceeded wait-time targets based on their designated priority levels declined by 100% and 55% for P2 and P3 cases, respectively, post-ACCESS (p < 0.0001). There was no significant change in the length of wait-times for elective cancer cases pre- and post-ACCESS. Additionally, the proportions by type of cancer treated at VH was significantly different post-ACCESS (p < 0.0001): esophageal, prostate, and gynaecological cancer cases declined by 19%, 20%, and 19%, respectively, while head and neck cancer, and lung cancer cases increased by 79% and 15%, respectively.

**Table 3:** Distribution of elective cancer operations performed by subspecialty surgical oncologists (non-general surgeons) at Victoria Hospital, before and after the implementation of ACCESS (pre- and post-ACCESS, respectively)

**Variable**	**Pre-ACCESS, n (%)**	**Post-ACCESS, n (%)**	**Change, n (%)**	**P value**
Number of cases, n	1685	1624	-61 (-4)	-
Number of cases by priority level, n (%)				<0.0001
P2	187 (11)	95 (6)	-92 (-49)	
P3	1027 (61)	768 (47)	-259 (-25)	
P4	471 (28)	761 (47)	+290 (+62)	
No. of cases exceeding wait-time targets by priority, n (%)				0.39
P2	120 (64)	61 (64)	-59 (-49)	
P3	485 (47)	297 (39)	-188 (-39)	
P4	122 (26)	118 (16)	-4 (-3)	
Median wait-times by priority, days (range)				0.52
P2	19 (1–215)	17 (1–55)	-2 (-10)	
P3	27 (0–274)	23 (0–108)	-4 (-14)	
P4	66 (0–246)	41 (0–207)	-25 (-37)	
Type of cancer, n (%)				< 0.0001
Gastric	21 (1)	10 (0.6)	-11 (-52)	
Endocrine	238 (14)	172 (11)	-66 (-28)	
Genitourinary (excluding prostate)	228 (14)	230 (14)	+2 (+1)	
Gynecological	350 (21)	284 (17)	-66 (-19)	
Head and neck (excluding thyroid)	154 (9)	276 (17)	+122 (+79)	
Lung	168 (10)	194 (12)	+26 (+15)	
Lymph	2 (0.1)	3 (0.2)	+1 (+50)	
Peripheral nervous system	1 (0.1)	3 (0.2)	+2 (+200)	
Prostate	132 (8)	105 (6)	-27 (-20)	
Skin carcinoma^1^	8 (0.5)	7 (0.4)	-1 (-13)	
Skin melanoma	49 (3)	30 (2)	-19 (-39)	

^1^Includes basal and squamous cell carcinoma.

## Discussion

As ACS continues to flourish around the world, an increasing number of studies have emphasized the benefits of this care model for patients with general surgical emergencies [2,5,8,15-18]. Surgical departments, however, have historically been expensive to run because of the costly equipment, support staff, as well as the specialized nursing and medical staff required [19]. The operating room, therefore, is viewed as a necessary but expensive liability in the financially-constrained Canadian healthcare system. Consequently, funding for the implementation of surgical programs such as ACS services often requires the reallocation of pre-existing operating room resources.

Prior to the implementation of ACCESS at our institution, there was no structured system for performing emergency general surgery cases during the daytime. Emergency patients would usually have their operation in the evening or night, after the completion of the daytime elective caseload, or they would have their operation during the daytime at the expense of cancelling one or more elective cases. Alternatively, patients would stay in the hospital—sometimes for days— before a surgeon was able to perform an operation during his elective schedule. The goal of ACCESS, therefore, was to provide more timely access to the OR for emergency general surgery, while decreasing the amount of expensive “after-hours” surgeries, all the while without increasing the overall general surgery operating volume.

The general surgeons at our institution agreed to participate in ACCESS for several reasons: they would be provided with 28 hours of operating time per week, almost two times more than their weekly allotted elective OR time; they would operate less at night because most emergency cases could be performed during the subsequent day; they would have significant control over their billing during ACCESS since they were paid by fee-for-service; and, most importantly, they would be able to focus on their elective practice and academic pursuits when they were not covering ACCESS. With respect to the latter, all emergency general surgery patients were admitted to ACCESS, even if they were operated by an on-call surgeon in the evening or night-time, thereby reducing the inpatient load for all non-ACCESS surgeons.

Since more than 50% of the dedicated OR time for ACCESS came from previous elective OR time, one of the concerns stemming from this reallocation was that there may be an impact on the timeliness of care for patients awaiting elective surgery, particularly for the treatment of cancer. Surgery is a key component of curative treatment for many cancers. Delays in cancer treatment can increase the risk of metastases, potentially precluding the opportunity for cure, as well as the risk of oncologic emergencies such as luminal obstruction [20,21]. Additionally, longer waits for cancer treatment can lead to significant psychological stress and anxiety in patients [20-24]. While surgical wait-times could be reduced by the provision of additional OR resources, the challenge faced by healthcare professionals and hospital administrators is to balance the medical and psychosocial costs of waiting against other demands on healthcare resources.

Initiatives such as the Ontario Wait Time Strategy have been implemented to ensure that wait times remain appropriate [10,12,14,25,26]. A fundamental component of this strategy was the development of the Wait Time Information System (WTIS) to collect wait-time data from hospitals throughout the province [26]. To complement the WTIS, the MOHLTC and CCO developed wait time targets for cancer surgery, based on evidence-based medicine and expert consensus [10,11]. CCO determined that most patients with suspected or confirmed invasive cancer could be assigned to a single priority category (P3). However, three additional categories (P1 for emergent cases, P2 for very aggressive tumours, and P4 for indolent tumours) were created to reflect the heterogeneity of tumour biology. Finally, using a “pay for performance” approach, hospital funding for surgical cancer care was tied to the achievement of wait-time milestones [11,13].

At VH, the impetus to reallocate general surgery operating resources to ACS was done as we felt this would help improve overall patient care. Another goal was to reduce the long stays in the emergency department for general surgery patients requiring emergency operations, without adversely affecting wait-times for elective cases. Along with the implementation of ACCESS at VH, the performance of cancer operations not requiring inpatient admission (such as breast cancer and melanoma) was shifted to a nearby ambulatory-care centre. During the study period, CCO also mandated a shift in the treatment of select malignancies (particularly hepatobiliary and colorectal cancer) away from community hospitals to high-volume tertiary-care centres such as VH. Consequently, there was a significant change observed in the composition of cancer surgeries performed at VH after the implementation of ACCESS, with fewer breast and melanoma surgeries, and increased proportions of colorectal and hepatobiliary cases.

Interestingly, we observed a significant change in the distribution of cancer cases by priority post-ACCESS, for all surgeons (including general surgeons) at Victoria Hospital: the proportion of P2 and P3 cases declined, while the proportion of P4 cases increased significantly. Since the general surgeons participating in ACCESS also perform cancer surgeries during their elective practices, they may have been performing P2 and P3 cancer cases on standby during ACCESS time (when there was a paucity of emergency general surgery cases), thereby contributing to the decline in P2 and P3 cases electively. If this was the case, surgeons may have had more time during their elective OR time to operate on patients with P4 cancers. This possible change may also partially explain the significant reduction in the number of general surgery cancer cases that exceeded the wait-time targets. Alternatively, surgeons at VH may have become more conservative in assigning priority levels for cancer patients in order to avoid missing wait-time targets and the associated penalties. This explanation may be more likely given the down-grading present across all surgical specialties at VH, although a case–control analysis of cancer patients may determine if this has been occurring since the implementation of the Wait Time Strategy. One of the limitations of this study was our inability to accurately determine the number of cancer surgeries performed during ACCESS time because standby cancer operations were usually reported as emergency cases rather than elective surgeries. With the recent integration of operative databases for emergency and elective cases at our institution, however, future prospective analyses may provide this important information.

Overall, there was no significant change in cancer surgery wait times pre- versus post-ACCESS. Therefore, the implementation of ACCESS, and the resultant reallocation of OR time from elective to emergency case loads, did not negatively impact wait times for elective cancer surgery. Additionally, wait-times remained unchanged despite the significant increase in the performance of hepatobiliary and colorectal surgeries post-ACCESS, which are typically longer and more complex than the breast cancer and melanoma cases that were moved off-site. Finally, while general surgery OR resources were redistributed to accommodate ACCESS at a single institution (VH), other hospitals within the city and region may have also adjusted their practices, contributing to keeping wait-times on target.

To our knowledge, this is the first study to examine the impact of implementing an ACS service on wait-times for elective surgeries. Miller *et al.*[27] and Barnes *et al.*[15] observed a 23% and 44% increase in operative productivity in terms of elective caseloads, respectively, but an overall decline in general surgery operative volumes because of a reduction in emergent cases [15]. However, neither study considered wait-times for elective cases. While many studies examining the impact of ACS services originate from the United States, American ACS services often differ significantly from Canadian models. In Canada, general surgeons participating in ACS services often also perform cancer operations as part of their elective practices, whereas many American acute care surgeons are trauma specialists who do not routinely perform oncological operations.

One of the limitations of this study is that the effect of ACCESS on wait-times for non-cancer elective operations, such as elective bowel resections for non-malignant pathology or hernia repair, was not explored. Because of the lack of organized databases to measure wait-times for elective non-cancer operations, it was difficult to ascertain the impact of ACCESS on wait-times for these cases. However, surgeons are given the discretion to book elective cases during ACCESS OR time if there are no emergency cases on the board. Most have reported excellent patient satisfaction with the development of “standby lists”, whereby patients who are booked for elective non-cancer surgeries are called into the hospital on the day of their operation. Additionally, as discussed earlier, the recent integration of elective and emergency operating databases, which also include non-cancer operations, may allow for future prospective studies to address this important issue.

In conclusion, the reallocation of operating room resources from elective surgical practice towards an ACS service did not appear to affect the timeliness of care provided to patients waiting for elective cancer surgeries, and thus such concerns should not serve as a barrier for centres considering implementing an ACS service.

## Competing interest

The authors do not have any actual or potential conflicts of interest to declare.

## Authors’ contributions

RVA, NP, and KL conceived and designed the study. RVA and KV collected the data and performed the statistical analysis. RVA drafted the manuscript. DP helped to draft the manuscript. RVA, DP, KV, SC, NP, and KL provided critical revisions of the manuscript for important intellectual content. All authors read and approved the final manuscript.
